# Application of positive airway pressure in restoring pulmonary function and thoracic mobility in the postoperative period of bariatric surgery: a randomized clinical trial

**DOI:** 10.1590/bjpt-rbf.2014.0054

**Published:** 2014

**Authors:** Patrícia Brigatto, Jéssica C. Carbinatto, Carolina M. Costa, Maria I. L. Montebelo, Irineu Rasera-Júnior, Eli M. Pazzianotto-Forti

**Affiliations:** 1 Faculdade de Ciências da Saúde, Universidade Metodista de Piracicaba (UNIMEP), Piracicaba, SP, Brazil; 2 Faculdade de Ciências Exatas e da Natureza, UNIMEP, Piracicaba, SP, Brazil; 3 Clínica Bariátrica de Piracicaba, Piracicaba, SP, Brazil

**Keywords:** bariatric surgery, physical therapy specialty, spirometry

## Abstract

**Objective:**

To evaluate whether the application of bilevel positive airway pressure in the postoperative period of bariatric surgery might be more effective in restoring lung volume and capacity and thoracic mobility than the separate application of expiratory and inspiratory positive pressure.

**Method:**

Sixty morbidly obese adult subjects who were hospitalized for bariatric surgery and met the predefined inclusion criteria were evaluated. The pulmonary function and thoracic mobility were preoperatively assessed by spirometry and cirtometry and reevaluated on the 1^st ^postoperative day. After preoperative evaluation, the subjects were randomized and allocated into groups: EPAP Group (n=20), IPPB Group (n=20) and BIPAP Group (n=20), then received the corresponding intervention: positive expiratory pressure (EPAP), inspiratory positive pressure breathing (IPPB) or bilevel inspiratory positive airway pressure (BIPAP), in 6 sets of 15 breaths or 30 minutes twice a day in the immediate postoperative period and on the 1^st ^postoperative day, in addition to conventional physical therapy.

**Results:**

There was a significant postoperative reduction in spirometric variables (p<0.05), regardless of the technique used, with no significant difference among the techniques (p>0.05). Thoracic mobility was preserved only in group BIPAP (p>0.05), but no significant difference was found in the comparison among groups (p>0.05).

**Conclusion:**

The application of positive pressure does not seem to be effective in restoring lung function after bariatric surgery, but the use of bilevel positive pressure can preserve thoracic mobility, although this technique was not superior to the other techniques.

## Introduction

In obese individuals, a combination of factors such as reduced lung and chest wall compliance, increased lung elastic resistance and exaggerated distension of the diaphragm may affect the respiratory system, resulting in decreased lung volumes and capacities. This condition is especially important considering that respiratory changes caused by abdominal surgery may be more pronounced in obese patients^1^^,^ 2. 

Bariatric surgery may impair the lung function during the postoperative period, expressed by reduced lung volumes and diaphragmatic and thoracoabdominal mobility^3^. The change in the pulmonary mechanics generates a restrictive pattern with reduced vital capacity (VC) and functional residual capacity (FRC). The VC and forced vital capacity (FVC) are usually reduced in the postoperative period to approximately 40-50% of the preoperative values, which persists for at least 10 to 14 days^4^. The use of anesthetics, neuromuscles bloquers and analgesics^5^^-^ 7, surgical trauma, loss of abdominal muscle integrity^8^, manipulation of the viscera, the consequent reflex inhibition of the phrenic nerve and postoperative pain^9^ all generate diaphragmatic dysfunction^5^^,^ 7, which peaks between two to eight hours after surgery^10^ and triggers a reduction in lung volumes and capacities, changes in the ventilation/perfusion ratio, decreased thoracoabdominal expansion^2^, inefficiency in the airway defense mechanisms, such as cough, and depression of the immune system, factors that increase the risk of developing respiratory complications such as atelectasis^11^^,^ 12, severe hypoxemia, pulmonary embolism, aspiration pneumonia and acute respiratory failure^2^^,^ 7 ^,^ 11 ^,^ 12 ^,^. 

Some evidence suggests that for these reasons, pre- and postoperative physical therapy follow-up of patients who undergo bariatric surgery is extremely important for preventing complications inherent to the surgical process and for recovering lung function^13^, whereas It has been suggested that the use of respiratory physical therapy in the postoperative routine is not justified because few clinical trials show its prophylactic effectiveness^14^. In 2012, Hanekom et al.^15^ concluded that, due to the poor quality of the available studies, there was still uncertainty regarding the value of physical therapy routinely performed to prevent pulmonary complications after abdominal surgery. Thus, well-designed studies on the subject that may contribute to establishing more effective physical therapy procedures to be performed in the postoperative period of abdominal surgery are needed^16^^,^ 17. 

Among the respiratory physical therapy techniques that aim to preserve or improve lung function, promoting growth or maintenance of lung volumes and capacities, are devices with positive airway pressure, which can be used in the postoperative period of thoracoabdominal surgery to prevent or treat hypoxic respiratory failure, improve arterial oxygenation, reduce atelectasis and decrease respiratory work without generating increased incidence of fistulas or dehiscence of surgical anastomoses^18^^-^ 21. 

The use of positive airway pressure has been shown to be effective in restoring the FRC and promotes changes in other lung volumes and capacities, such as inspiratory reserve volume (IRV), expiratory reserve volume (ERV) and FVC. However, there is controversy regarding the maintenance produced by positive airway pressure^4^. EPAP (Expiratory Positive Airway Pressure), BIPAP (Bilevel Positive Airway Pressure) and intermittent positive pressure breathing (IPPB) are techniques using positive airway pressure aimed at lung re-expansion, preventing premature airway collapse and thus preventing pulmonary atelectasis, and may contribute to the reduction in postoperative complications of the pulmonary restrictive syndrome associated with obesity^22^^-^ 24. The EPAP technique uses only positive end-expiratory pressure (PEEP), reducing the expiratory flow^22^, whereas BIPAP combines PEEP with the benefits of support pressure, and they can be adjusted to keep the lung expanded throughout the respiratory cycle, promoting lung inflation^23^^,^ 24. The IPPB technique allows, in addition to alveolar recruitment, the synchronization of inspiratory time, aiming to reduce the respiratory effort and resume normal lung function^10^. 

Considering that the obese population may have restrictive lung characteristics, which may be even more pronounced during the abdominal postoperative period, and that positive airway pressure techniques may be able to restore lung function and chest mobility differently, in this study, three respiratory techniques were investigated: the IPPB technique which favors the inspiratory capacity (IC) by promoting positive airway pressure only in the inspiration phase; the positive expiratory pressure generated by EPAP which, mainly favors the FRC in an attempt to promote the maintenance or recovery of ERV; and, BIPAP, which provides positive pressure in both phases of the respiratory cycle, and tends to favor VC, encompassing the benefits of the other two techniques. Thus, the objective of this study was to test whether the application of bilevel positive airway pressure during the postoperative period of bariatric surgery would be more effective than applying inspiratory and expiratory positive pressures separately in restoring lung volumes, lung capacities, and thoracic mobility. 

## Method

### Experimental design 

This clinical trial was developed respecting the rules of conduct in experimental research with humans after being approved by the Ethics in Research Committee of the Universidade Metodista de Piracicaba (UNIMEP), Piracicaba, São Paulo, Brazil, under approval no. 89/12 and registered at Clinicaltrials.gov under identifier NCT01872663. 

The sample size calculation was based on a pilot study, in which the mean (0.13) and standard deviation (0.17) of the differences between pre- and postoperative ERV values were obtained using the ANOVA test in the BioEstat 5.3 application, adopting a statistical power of 90% and an alpha of 0.05. Thus, the number of 17 volunteers per group was determined. 

### Participants 

In total, 68 morbidly obese adult women admitted to a hospital in the city of Piracicaba, São Paulo, Brazil for elective bariatric surgery by the responsible physician, with a prescription for respiratory physical therapy. The subjects had to meet the following inclusion criteria: body mass index (BMI) between 40 and 55 kg/m^2^, aged between 25 and 55 years, candidates for Roux-en-Y gastric bypass bariatric surgery by laparotomy, nonsmokers, with chest x-ray and preoperative pulmonary function test within the parameters of normality and who signed the consent form. Subjects with asthma, chronic obstructive pulmonary disease (COPD) or obstructive sleep apnea (OSA) syndrome were excluded, as were subjects who presented with hemodynamic instability, hospital stay longer than three days, presence of postoperative complications or an inability to understand or refusal to perform the evaluations or the proposed treatment. 

### Procedures 

The lung function and chest mobility of the patients were evaluated preoperatively, immediately after hospital admission, and reassessed on the first postoperative day, after the physical therapy sessions were completed. The investigator who conducted the assessments was blind to the treatment received, and the investigator who performed the treatments was blind to the assessments. During the preoperative evaluation, the presence of comorbidities such as systemic arterial hypertension (SAH), diabetes mellitus and dyslipidemia was recorded. 

**Figure 1:**
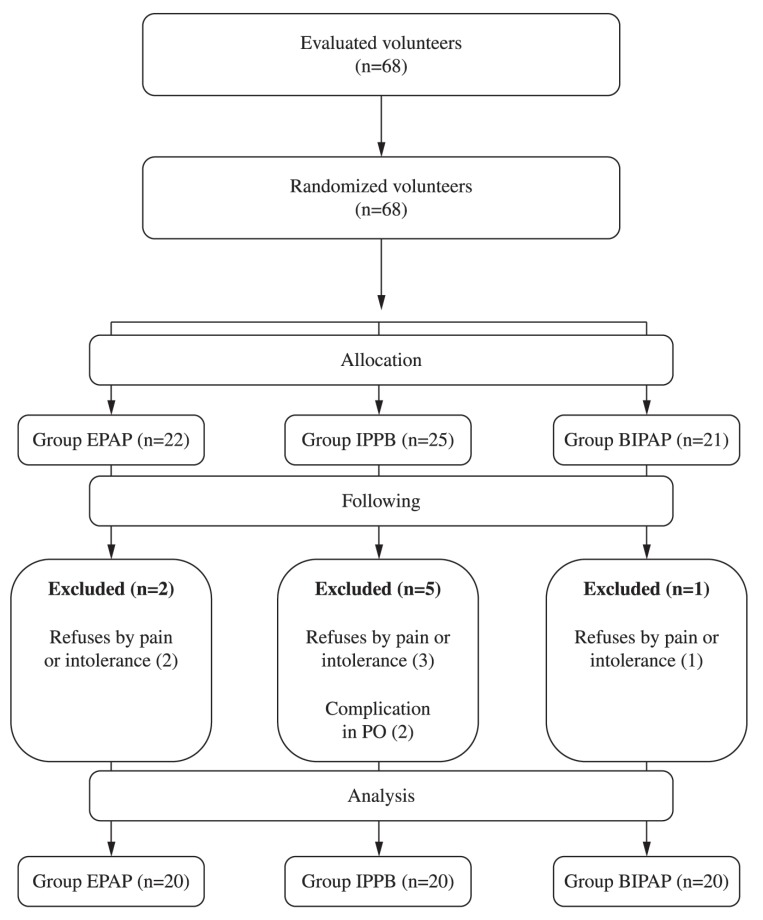
Flow diagram of the study sample. EPAP: Expiratory Positive Airway Pressure; IPPB: Intermittent Positive Pressure Breathing; BIPAP: Bilevel Positive Airway Pressure. PO - Post Operatively.

After the preoperative evaluation, the 68 volunteers were allocated into three groups using a block randomization process in Microsoft^(r)^ Excel 2007 conducted by an investigator blind to both the clinical data and the volunteers' assessment. The groups were as follows: Group EPAP (Expiratory Positive Airway Pressure), Group IPPB (Intermittent Positive Pressure Breathing) and Group BIPAP (Bilevel Positive Airway Pressure). During the application of interventions, eight subjects were excluded. In the end, a total of 60 subjects comprised three groups of 20 (Figure 1). 

All subjects received the proposed intervention twice a day in the immediate postoperative period and on the first postoperative day (1^st^ PO); in combination with positive airway pressure therapy. All subjects also received conventional physical therapy, also performed twice a day postoperatively, consisting of exercises with diaphragmatic breathing, deep inspirations, fractionated inspirations, breathing exercises associated with upper limb movement^25^^,^ and incentive spirometer. One series with 15 repetitions was developed for each exercise, with a mean duration of 20 to 30 minutes, in addition to exercises to prevent deep vein thrombosis and to promote ambulation. 

Lung function was assessed by spirometry of the subjects, performed using a microQuark computerized ultrasonic spirometer, USB model (Cosmed^(r)^, Rome, Italy). The slow vital capacity (SVC) maneuver was performed according to the guidelines of the American Thoracic Society (ATS) and the European Respiratory Society (ERS)^26^ and repeated until three acceptable and two reproducible curves were obtained, not exceeding more than eight attempts. To calculate the predicted values, the equation proposed by Pereira et al.^27^ for the Brazilian population was used, and the values of SVC, ERV, IRV and tidal volume (TV) were selected according to the recommendations of Pereira^28^. The IC values were calculated from the sum of IRV and TV. 

The thoracic mobility evaluation was performed by cirtometry at the axillary and xiphoid levels by the same researcher, using a tape measure scaled in centimeters, measuring the circumferences after a maximal inspiration and after a maximal expiration while the subjects were in a standing position. Three measurements were repeated at each level, and the maximum value obtained during inspiration and the minimum value obtained during expiration were computed. The absolute difference between these values was considered to represent the thoracic mobility for each level^25^^,^ 29. 

To minimize the interference of pain on postoperative assessments, before initiating the assessments, the subjects rated their pain level using a Visual Analogue Scale (VAS)^30^^,^ 31. When the pain was rated above 4, the nursing staff was requested to administer an analgesic with dipyrone, according to medical prescription, and the pain was then scored again after 30 minutes before the revaluation was started. 

The EPAP was applied using a silicon face mask with a one-way valve (Seal Flex Multi-Strap, Respironics, Ireland, USA) and a spring loaded PEEP valve (Vital Signs Inc., Totoma, New Jersey, USA) set to 10 cmH_2_O^3^, which was positioned and fixed by the physical therapist on the face of the subject to prevent air leakage. The subjects were instructed to perform six series of 15 breaths, with average-amplitude nasal inspiration and expiration against the resistance provided by the PEEP valve^32^, emphasizing diaphragmatic breathing. The volunteers rested for 1-2 minutes between each series, and each session lasted approximately 20 minutes. 

The IPPB was applied using a Müller Reanimator device (Engesp^(r)^, Curitiba, Paraná, Brazil) with an endotracheal pressure of 20-30 cmH_2_O corresponding to 2-3 kgf/cm^2^ in the oxygen pressure regulator valve, and physiological saline was used as the diluent in the micronebulizer^21^^,^ 33. The subjects were instructed to inspire into the device nozzle on the command of the physical therapist simultaneously with the triggering of the equipment, to sustain the inspiration for 1-2 seconds, and then to breathe freely. Six series of 15 respiratory cycles were performed during each session, with 1-2 minutes of rest between each series, with each session lasting approximately 30 minutes. 

The noninvasive application of BIPAP was performed using the VPAP(tm) III ST-A device (Resmed^(r)^, San Diego, California, USA) connected to a simple facemask with an inflatable edge by a corrugated trachea and attached to the face of the subject by a rubber retainer. The EPAP was set to 8 cmH_2_O, and the inspiratory positive airway pressure (IPAP) was initially set to 12 cmH_2_O^34^ and readjusted to maintain a respiratory rate between 12 and 20 breaths per minute with a TV of approximately 8 to 10 mL/kg of ideal weight, calculated by the formula 45.5 + 0.91 (height - 152.4)^35^. During the 30 minutes of application, the subjects were instructed to perform nasal inspiration and oral expiration. 

Throughout the application of the techniques, the subjects remained in the 45º Fowler position, and the investigator remained beside them and monitoring vital signs and respiratory comfort. 

### Statistical analysis 

The statistical analysis of data was performed using the software R version 3.0.1, and the normality of data distribution was verified using the Shapiro-Wilk test. 

For the intragroup analysis of spirometric variables and preoperative and postoperative thoracic mobility, Student's t-test for paired samples or the Wilcoxon test was performed. For intergroup comparison, the values of the differences between the pre-and postoperative period were analyzed by ANOVA or Kruskal-Wallis tests. 

A significance level of 5% was adopted for all analyses. 

## Results

Table 1 shows the results of age, anthropometric characteristics and comorbidities of the study subjects allocated to the groups according to the proposed treatment. 

**Table 1:** Age, anthropometric characteristics (values in mean and standard deviation) and comorbidities of study subjects.

	Group EPAP (n=20)	Group IPPB (n=20)	Group BIPAP (n=20)
**Age** (years)	38.85±8.42	38.70±8.59	40.60±8.78
**Weight** (kg)	114.99±17.96	110.13±14.82	113.69±16.08
**Height** (m)	1.59±0.06	1.61±0.06	1.61±0.09
**BMI** (kg/m^2^)	45.39±6.82	42.39±5.36	44.34±8.14
**Hypertension** (n)	12	10	13
**Diabetes Mellitus** (n)	6	3	6

BMI: body mass index. EPAP: Expiratory Positive Airway Pressure; IPPB: Intermittent Positive Pressure Breathing; BIPAP: Bilevel Positive Airway Pressure.

There was no difference among groups for the variables age, weight, height, BMI, presence of SAH or presence of diabetes mellitus. 

Table 2 shows the spirometric variables of the SVC maneuver of each group for the evaluations before and after surgery in absolute values and as percentages of predicted SVC and ERV values. A significant decrease was observed for all variables in the postoperative period compared to the preoperative period. Table 1 also shows decreases between the pre- and postoperative values in percentages, and when compared with each other, these values were not significantly different. 

**Table 2:** Absolute values and percentages of predicted spirometric variables in the SVC maneuver for each group pre- and postoperatively, expressed as the mean and standard deviation.

	Group EPAP (n=20)				Group IPPB (n=20)		Group BIPAP (n=20)		
	PRE	POST	DIF (%)	PRE	POST	DIF (%)	PRE	POST	DIF (%)
SVC (L)	2.92±0.62	1.86±0.46*	36.30	3.24±0.51	2.31±0.33*	28.70	3.11±0.68	2.11±0.59*	32.15
SVC (% pred)	88.29±13.15	56.74±14.33	35.74	96.37±14.43	68.58±10.12	28.83	93.19±13.49	63.90±17.08	31.43
ERV (L)	0.51±0.21	0.27±0.14*	47.06	0.48±0.31	0.32±0.18*	33.33	0.51±0.34	0.32±0.27*	37.25
ERV (% pred)	45.56±15.44	24.42±11.76	46.39	42.10±27.11	28.61±14.52	32.04	45.17±26.63	27.65±21.81	38.79
IRV (L)	1.53±0.56	0.97±0.43*	36.60	2.01±0.60	1.36±0.29*	32.34	1.73±0.71	1.20±0.43*	30.64
TV (L)	0.91±0.39	0.62±0.21*	31.87	0.76±0.26	0.63±0.18*	17.11	0.88±0.27	0.60±0.26*	31.82
IC (L)	2.44±0.61	1.59±0.47*	34.84	2.77±0.45	1.98±0.31*	28.52	2.61±0.50	1.80±0.43*	31.03

SVC: slow vital capacity; ERV: expiratory reserve volume; % pred: percentage of predicted; IRV: inspiratory reserve volume; TV: tidal volume; IC: inspiratory capacity; DIF: difference between pre and postoperative;

* significant difference between pre and postoperative (p<0.05). L = liters, pre =preoperatively, post = post-operatively.

Table 3 shows the values of thoracic mobility measures at the axillary and xiphoid levels for the groups pre- and post-surgery. The intragroup analysis showed a significant decrease in the axillary and xiphoid mobility for the EPAP and IPPB groups postoperatively; for the BIPAP group, no significant differences were found. When comparing the differences of pre- and postoperative thoracic mobility values, there was no difference between the levels evaluated, regardless of the treatment received. 

**Table 3:** Thoracic mobility values in the axillary and xiphoid levels for each group pre- and postoperatively, expressed as the mean and standard deviation.

	AXILLARY (cm)			XIFOIDEANA (cm)		
	PRE	POST	DIF (%)	PRE	POST	DIF (%)
Group EPAP (n=20)	3.70±1.20	2.50±1.05*	32.43	2.55±1.11	1.35±0.84*	47.06
Group IPPB (n=20)	3.93±1.31	2.88±0.79*	26.72	2.78±1.25	1.53±0.82*	44.96
Group BIPAP (n=20)	3.75±1.73	2.78±1.08	25.87	2.40±1.73	1.60±0.79	33.33

DIF: difference between pre and post;

* significant difference between pre and postoperative (p<0.05). Pre = preoperatively, post = postoperatively, dif (%) = percentage difference btwn. pre- & postoperatively.

## Discussion

The main results of this study showed a significant decrease in spirometric variables during the postoperative period, regardless of the technique used, and preservation of thoracic mobility only in volunteers from the BIPAP group. 

The decreased pulmonary function after surgical procedures can be explained by factors inherent to the procedure itself, such as the use of anesthetics and analgesics, the loss of integrity of the abdominal muscles and the consequent decrease in muscle contraction force and diaphragmatic dysfunction, as well as by factors that interfere in performing spirometric maneuvers, such as pain and fear of deep inspiration^6^^,^ 36. In this study, there was concern about assessing pain and requesting the administration of analgesics according to medical prescription before the postoperative evaluations, when necessary, to prevent the results from being affected by this factor. However, we suggest that the effects of the surgical procedure, associated with obesity, were important contributors to the significant decrease in spirometry and thoracic mobility values in the three groups. 

The excess fat stored in the abdominal cavity exerts a direct mechanical effect on the ribcage and on the diaphragm, restricting chest expansion, with a consequent decrease in lung volumes^37^^-^ 39. This chest wall restriction is greater when the obese patient is in the supine position, such as during surgery or during the hospitalization period, causing major muscle overload for ventilation and resulting in dysfunction of the respiratory muscles^40^^,^ 41. 

The decreased chest wall compliance due to increased abdominal pressure, administration of anesthetics and postoperative pain may cause a prolonged reduction in lung volumes and capacities. The BIPAP therapy is believed to reverse these phenomena through the combined positive PEEP effects and inspiratory support pressure, allowing the recruitment of collapsed alveoli zones, increasing pulmonary ventilation and improving gas exchange, in addition to increasing chest expansion^9^^,^ 23 ^,^ 42 ^,^ 43. However, in this study, BIPAP was not able to restore lung function postoperatively, and its effectiveness was only observed in the restoration of the thoracic mobility when compared to the other positive the therapy exerted a dose and time dependent pressure techniques used. effect, demonstrating better results when higher 

Pessoa et al.^18^ used the BIPAP technique in the pressure levels were used for prolonged times. immediate postoperative period of bariatric surgery, Considering this finding, we suggest that more still in post-anesthetic recovery, and observed that significant results were not found in this study because the technique was applied for short periods, in 30-minute sessions. 

The absence of significant positive effects when using the positive pressure techniques in this study can also be explained by the time that the techniques were applied, as they were only begun approximately four hours after the end of surgery. Forgiarini Junior et al.^43 ^demonstrated that physical therapy, when initiated in the post-anesthetic recovery room, might be beneficial for patients who underwent abdominal surgery because the pulmonary function values in patients who received physical therapy earlier had lower variation in postoperative spirometry values compared to the preoperative values than the group that started physical therapy on the ward. 

According to the literature, general anesthesia may worsen hypoventilation during the early hours of postoperative recovery due to increased alveolar instability during this period, and the early application of positive pressure might be able to improve alveolar ventilation in areas that might have collapsed during the surgical procedure^44^^-^ 47. In the post-anesthesia recovery room, the patient's tolerance was considered to be facilitated by the residual sedative effect of anesthetics and opioids administered for analgesia^23^, allowing the techniques to be applied over an extended period of time, which was not performed in this study because the subjects were already in the hospital room. 

Another important factor to be considered in this study as having a likely effect on the results, especially regarding lung function, was the time of the postoperative reassessment. The volunteers were reassessed approximately 36 hours after surgery, and this time might not have been sufficient for restoring lung volumes and capacities regardless of the technique applied because until that time, the diaphragmatic function was not completely restored. In the study by Paisani et al.^47^, which evaluated the pattern of lung volumes and capacities of patients in the first postoperative period after gastroplasty, decreases of 30 to 50% were observed in the values of the variables compared to their pre-operative values, and on the fifth postoperative day, the VC had not yet returned to its initial values. In the present study, the lung volumes and capacities, also reassessed on the first postoperative day, decreased by 17 to 46%, and the variables did not recover before hospital discharge. 

In a study by Barbalho-Moulim et al.^3^, EPAP was unable to prevent the reduction in thoracic mobility at the axillary and xiphoid levels and of TV and IRV measures, as also occurred in the present study, possibly because EPAP is a technique that does not stimulate inspiratory "sighs" but instead is associated with low lung volumes and reduced expiratory flow. 

According to Müller et al.^33^, the IPPB technique allows synchronism between the operator and the patient, respecting the respiratory cycle, promoting better adaptation to the device and preventing respiratory distress, and it was thus considered an effective technique for TV gain and hence for lung re-expansion. However, these beneficial effects were not observed in the present study. 

We suggest that techniques with positive pressure have similar effects regarding the restoration of lung volumes and capacities and chest expansion during the postoperative period of bariatric surgery, regardless of being applied during inspiration, expiration or both, and are not effective when applied according to the protocol established for this study, indicating that during the early postoperative days, lung function remains impaired by the obesity effects associated with abdominal surgery effects. 

All subjects underwent the same surgical technique by the same surgical team with similar surgery time and anesthesia duration, and during anesthesia, they remained on mechanical ventilation with ventilation parameters standardized by the medical team responsible, which were therefore not considered factors affecting the assessments in this study. 

Despite the small effect of positive pressure on pulmonary function and chest mobility of the subjects studied, it is important to note that the techniques applied did not cause any adverse effect or postoperative complications, such as fistula, abdominal distention or dehiscence of the surgical anastomosis. Thus, the present study considered the application of positive pressure during the postoperative period of bariatric surgery to have been safe. 

The short hospital stay of subjects who underwent elective bariatric surgery, who were discharged early in the second postoperative day, was considered a limitation of this study because it precluded reassessment at a later time. 

## Conclusion

The application of bilevel positive airway pressure within the protocol established in this study seems not to be effective in restoring lung volumes and capacities during the postoperative period of bariatric surgery. 

Better thoracic mobility results were obtained with the application of bilevel positive pressure than when positive inspiratory or expiratory pressure was applied separately, but this technique was not statistically more effective than the other techniques. 
